# Supplementary material for: Asymmetric Synthesis of Quaternary Hydantoins via a Palladium-Catalyzed Aza-Heck Cyclization
Source: J Am Chem Soc. 2025 Nov 14;147(49):44692–8. doi: 10.1021/jacs.5c16022 (PMC12703750; doi:10.1021/jacs.5c16022)
Supplement: Supplementary file 2 [file ja5c16022_si_002.zip › All NMR FID Files/S43/S43_AllNMR/1/pdata/1/email_EM04-044-1_1_1.pdf]

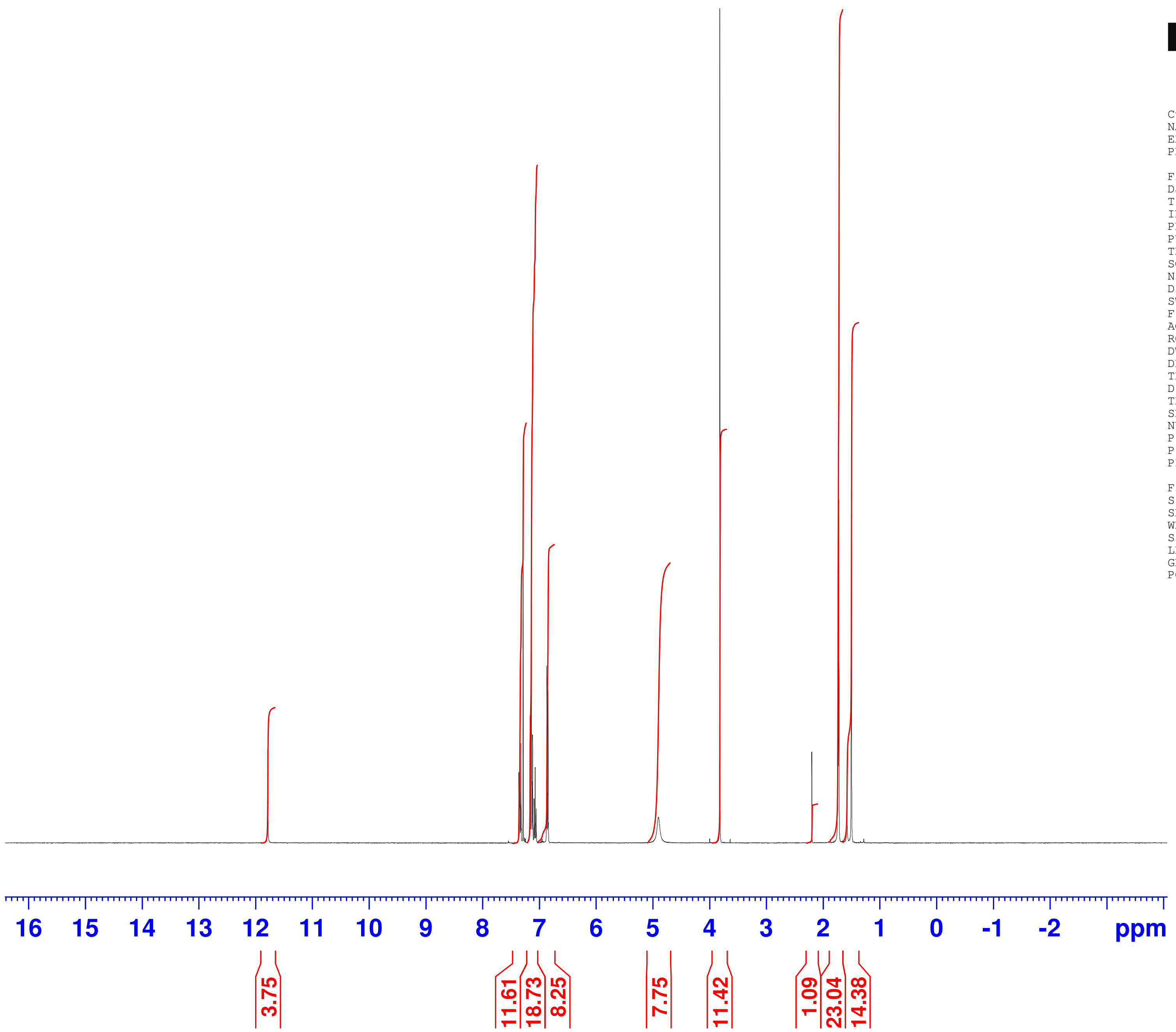


```
Current Data Parameters
NAME          EM04-044-1
EXPNO                  1
PROCNO                 1

F2 - Acquisition Parameters
Date_           20230401
Time               13.17 h
INSTRUM  Avance Neo 400 U of DE Brown Lab
PROBHD   Z824801_0171 (
PULPROG             zg30
TD                 65536
SOLVENT            CDCl3
NS                    16
DS                     2
SWH             8196.721 Hz
FIDRES          0.250144 Hz
AQ             3.9976959 sec
RG                   101
DW                61.000 usec
DE                 13.54 usec
TE                 298.2 K
D1            1.00000000 sec
TD0                    1
SFO1         400.1324708 MHz
NUC1                  1H
P0                  3.33 usec
P1                 10.00 usec
PLW1         16.44099998 W

F2 - Processing parameters
SI                 65536
SF           400.1300000 MHz
WDW                   EM
SSB                    0
LB                  0.30 Hz
GB                     0
PC                  1.00
```
